# Autophagy Regulators in Cancer

**DOI:** 10.3390/ijms241310944

**Published:** 2023-06-30

**Authors:** Juan Zhang, Qian Xiang, Man Wu, Yuan-Zhi Lao, Yan-Fang Xian, Hong-Xi Xu, Zhi-Xiu Lin

**Affiliations:** 1 School of Chinese Medicine, Faculty of Medicine, The Chinese University of Hong Kong, Hong Kong SAR 999077, China; juanzhang@cuhk.edu.hk (J.Z.); lisaxian@cuhk.edu.hk (Y.-F.X.); 2 School of Pharmacy, Shanghai University of Traditional Chinese Medicine, Shanghai 201203, China; xqianq1@163.com (Q.X.); wuman8938@126.com (M.W.); laurence.ylao@gmail.com (Y.-Z.L.); 3 Shuguang Hospital, Shanghai University of Traditional Chinese Medicine, Shanghai 201203, China; 4 Hong Kong Institute of Integrative Medicine, Faculty of Medicine, The Chinese University of Hong Kong, Hong Kong SAR 999077, China

**Keywords:** autophagy, cancer, inducers, inhibitors, apoptosis, metastasis, cancer therapy

## Abstract

Autophagy plays a complex impact role in tumor initiation and development. It serves as a double-edged sword by supporting cell survival in certain situations while also triggering autophagic cell death in specific cellular contexts. Understanding the intricate functions and mechanisms of autophagy in tumors is crucial for guiding clinical approaches to cancer treatment. Recent studies highlight its significance in various aspects of cancer biology. Autophagy enables cancer cells to adapt to and survive unfavorable conditions by recycling cellular components. However, excessive or prolonged autophagy can lead to the self-destruction of cancer cells via a process known as autophagic cell death. Unraveling the molecular mechanisms underlying autophagy regulation in cancer is crucial for the development of targeted therapeutic interventions. In this review, we seek to present a comprehensive summary of current knowledge regarding autophagy, its impact on cancer cell survival and death, and the molecular mechanisms involved in the modulation of autophagy for cancer therapy.

## 1. Introduction

Macroautophagy (referred to as autophagy throughout this review) is a highly conserved biological process that involves the engulfment of unfolded proteins or damaged organelles by double-membrane cytosolic vesicles known as autophagosomes that then get delivered to the lysosome for breakdown. Subsequently, the resulting macromolecules are released back into the cytosol for reuse [1,2].

As a self-eating process that is conserved from yeasts to humans, autophagy plays an important role in cancer. Its impact on tumorigenesis can vary depending on the context, exhibiting tumor-suppressive, tumor-promoting, or neutral effects [3,4]. In pre-malignant lesions, several studies have suggested that enhancing autophagy might prevent cancer development. However, in advanced cancers, both autophagy enhancement and inhibition have been implicated as therapeutic strategies [5,6]. Under physiological conditions, autophagy proceeds at basal levels in most tissues to ensure the routine turnover of superfluous or damaged components, maintain cellular homeostasis, and enable adaptation under stress. Moreover, autophagy can protect cells from becoming cancerous by removing reactive oxygen species (ROS), long-lived proteins, and damaged mitochondria.

Three major types of autophagy have been identified in mammalian cells according to the pathway to deliver the cargo: macroautophagy, microautophagy, and chaperone-mediated autophagy (CMA) [6,7]. Among these, macroautophagy is considered the primary form and has received more extensive research attention compared to microautophagy and CMA [8,9]. The term “autophagy” (intracellular degradation process) was coined by Christian de Duve in 1963, and since then, increasing research efforts have focused on understanding the relationship between autophagy and various diseases. Dysregulated autophagy has been implicated in a wide range of pathologies, including metabolic diseases, neurodegenerative diseases, infectious diseases, and cancers [10,11]. Microautophagy, a non-selective lysosomal degradative process, contributes to the development of human diseases such as neurodegenerative diseases, albinism, autosomal recessive diseases, and glycogen storage disease [7,12,13,14]. On the other hand, CMA stands out for its selectivity in targeting specific substrates for degradation. It plays an important role in regulating neuronal survival, the growth of tubular kidney cells, and the impact of NF-κB-mediated transcription in response to nutritional stress [6,15,16,17,18].

In recent years, the role of autophagy in cancer attracted increasing attention, and understanding its roles in cancer development may provide a new perspective for developing novel therapeutics for cancer treatment.

## 2. Autophagy and Its Formation Process

**Figure 1:**
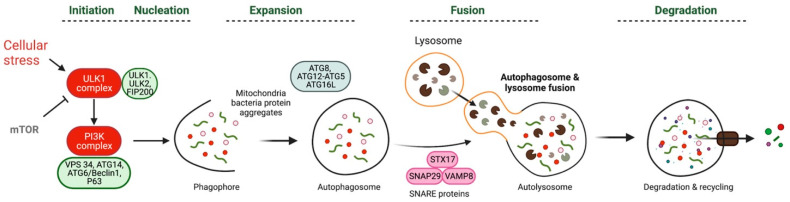
The process of autophagy. Autophagy triggered by cellular stress and regulated by mammalian target of rapamycin (mTOR-negative regulation). It involves the formation of protein complexes like unc-51-like autophagy activating kinase (ULK1) and phosphoinositide 3-kinase (PI3K). The autophagosome membrane undergoes expansion and elongation via ATG12 conjugation to ATG5. Autophagosomes eventually fuse with lysosomes with SNARE proteins for content degradation and macromolecule recycling.

Autophagy is tightly controlled via complex signaling pathways and core autophagy proteins. Autophagy mainly includes the following four stages in mammals: initiation, elongation, autophagosome formation, fusion with the lysosome, and degradation of the autophagic body [6,19] (Figure 1). Great efforts were made to identify the autophagy-related (ATG) genes in yeast in the 1990s [20,21], and approximately 40 ATG genes, of which its products are required for autophagy, have been identified in the yeast *Saccharomyces cerevisiae*.

### 2.1. Initiation of Autophagy

The process of initiation involves the induction of the phagophore formation. While most autophagic activity occurs at the basal level, it is upregulated in response to various environmental stresses [22]. Autophagy can be triggered via intra- or extracellular stresses, including hypoxia, oxidative stress, pathogen infection, and notably nutrient starvation [23,24,25]. The dynamic process of autophagy begins with the sequestration of cytoplasmic constituents by an expanding membrane called a phagophore or isolation membrane. Two major protein complexes play a role in this initiation step. The first is the class III PI3K complex, which includes VPS34 (also known as PIK3C3), ATG14, ATG6/Beclin1, and UV radiation resistance-associated gene protein (UVRAG, also known as p63) [26,27,28]. The second complex is the ULK1 (ATG1) complex, consisting of ULK1, ULK2 (mammalian homologs of ATG1), and FIP200 (also known as RB1CC1), an essential positive regulator of autophagosome formation. Upon stimulation, the ULK1 complex is activated, subsequently activating the class III PI3K complex [6,29,30]. The kinase of mTOR, a member of the PI3K-related kinase family, plays a negative regulatory role in the autophagy process in mammals [4,31,32]. Under nutrient-rich conditions, mTORC1 phosphorylates the ULK1 complex and ATG13, leading to the dissociation of the ULK1/ATG13/FIP200 complex and the inhibition of autophagy [33,34]. The nucleation and recruitment of the initial phagophore membrane require the class III PI3K complex, which, in turn, recruits two interconnected ubiquitin-like (Ubl) conjugation systems to the phagophore [35].

### 2.2. Elongation and Autophagosome Formation

The phagophore engulfs portions or organelles of the cytoplasm to form an autophagosome, which is a double-membrane sequestering vesicle [33,36,37,38,39]. Autophagosome formation involves multiple complex processes with several ATG proteins being recruited to govern this process [38]. Two Ubl conjugation systems, ATG8 and ATG12, mediate this process. ATG8 is conjugated to the lipid phosphatidylethanolamine (PE), while Atg12 is conjugated to ATG5. The conjugation of ATG12 to ATG5 involves the E1-like enzyme ATG7, which catalyzes the conjugation, and ATG10, an E2 conjugating enzyme. The resulting ATG12–ATG5 complex, an important regulatory molecule in the early stages of autophagosome formation, then interacts with ATG16 to form a multimeric complex. This ATG12–ATG5–ATG16 complex is associated with the growing phagophore and forms a larger multimeric complex via interaction with ATG16L [21,40,41]. The other Ubl conjugation system involved in autophagosome formation also relies on the E1-like enzyme ATG7. In this system, ATG7 activates ATG8, which is then transferred to the E2-like enzyme ATG3. ATG8 is subsequently linked to the target lipid PE via an amide bond, facilitated by the E3-like ATG12–ATG5 conjugate. Before interacting with PE, ATG8 must undergo processing via ATG4, a cysteine protease, which exposes a glycine residue. ATG4 also cleaves ATG8-PE to release free ATG8 after autophagosome formation [42,43]. Microtubule-associated protein light chain 3B (LC3B), the mammalian homolog of yeast ATG8, was identified as a protein that co-purified with microtubule-associated protein 1A and 1B from rat brains [44]. It serves as a marker protein for autophagy. LC3B is cleaved via ATG4 to form LC3B-I, which is then transferred to ATG3, then conjugated with PE to generate processed LC3B-II, which is an important maker of autophagy [45,46,47].

### 2.3. Fusion with the Lysosome and Degradation of the Autophagic Body

Once the autophagosome formation is completed, LC3B-II, which is attached to the outer membrane, is cleaved from PE via ATG4 and returns to the cytosol. The double-membrane autophagosome engulfs and sequesters intracellular components and then fuses with lysosomes (or vacuoles in yeast) to form an autophagolysosome (AL) [48,49]. During the peak of autophagy, numerous lysosomes are incorporated into autolysosomes. The fusion of autophagosomes with lysosomes requires SNARE proteins such as STX17, SNAP29, and VAMP8. STX17 translocates to the outer membrane of the completed autophagosome and promotes fusion by binding to SNAP29 and VAMP8 [6,49,50]. UVRAG, associated with the PtdIns3K complex, has also been reported to activate the GTPase RAB7 and facilitate fusion [51,52,53]. As the autophagosome fuses with the lysosome, it transforms into an autolysosome. Subsequently, the inner membrane disintegrates and is degraded together with sequestrated materials in acidic autolysosomes via hydrolase.

The lysosome, often described as a “cellular garbage can”, plays a key role in the autophagic process. The unique pH of the lysosome leads to degradation, a process relying on a series of lysosomal/vacuolar acid hydrolases, containing proteinases A and B (encoded by PEP4 and PRB1, respectively), the lipase ATG15, and cathepsin B, D (a homolog of proteinase A), and L in mammalian cells [54]. Research has shown that TFEB, a transcription factor, controls lysosomal biogenesis and regulates autophagy by driving the expression of related genes [55].

Therefore, to comprehensively understand the role of autophagy in cancer cells, it is necessary to explore the specific steps and regulatory mechanisms of the autophagy pathway. Examining the involvement of autophagy-related genes, signaling pathways, and selective autophagy processes can provide valuable insights into how autophagy impacts cancer development, progression, and therapeutic responses.

## 3. Autophagy in Cancer Development

**Figure 2:**
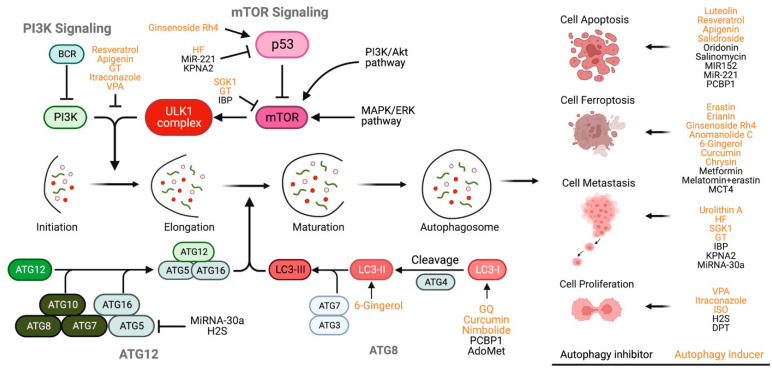
Mechanisms of autophagy regulators at indicated phases of autophagy. Schematic illustration showing that several interventions are available to induce or inhibit autophagy at the indicated phases. Also, the figure shows the effect of autophagy inducers or inhibitors in different cancer cellular processes, such as apoptosis, ferroptosis, metastasis, etc.; for additional details, please refer to Table 1 and Table 2.

Autophagy plays diverse roles and regulates multiple processes involved in cancer development, including cell apoptosis, cell ferroptosis, cell metastasis, and cell cycle. However, the role of autophagy in cancer development and progression is two-fold. On one hand, autophagy acts as a suppressor pathway, preventing tumor initiation. On the other hand, autophagy serves as a survival pathway by mitigating cellular metabolic stress, thereby contributing to tumor growth and progression [56]. The role and the molecular mechanisms of autophagy regulators at different stages of autophagy are summarized in Table 1 and Table 2, while Figure 2 illustrates these mechanisms.

### 3.1. The Role of Autophagy in Apoptosis

Autophagy is closely linked to cell death and apoptosis [57]. Its complex relationship with apoptosis has sparked controversy, as autophagy has been found to have the ability to inhibit, delay, or promote apoptosis [58]. In particular, under stressful conditions, autophagy acts as a crucial cytoprotective mechanism that enhances cell survival. Autophagy has been demonstrated to protect cells from various forms of cellular damage, including hypoxia, metabolic stress, detachment-induced anoikis, and apoptosis or necrosis induced by anticancer therapies or other cell death triggers [59]. Moreover, autophagy’s role in protecting cancer cells from apoptosis is well established, leading to extensive preclinical and clinical studies exploring the use of autophagy inhibitors in combination with other anticancer agents to enhance tumor cell death [57].

#### 3.1.1. Autophagy Inducers Regulate Cancer Cell Apoptosis

Luteolin, derived from *Trachelospermum jasminoides,* apocynaceae family, was found to induce autophagy and inhibit cell viability while promoting apoptosis in human liver cancer cells (SMMC-7721) [56]. Another compound, 7-O-geranylquercetin (GQ), a derivative of quercetin, induced autophagy and apoptosis via ROS generation in human non-small cell lung cancer cell lines (A549 and NCI-H1975). GQ treatment increased autophagosome formation and expression of autophagy-related proteins and suppressed p62 levels. The inhibition of autophagy reversed GQ-induced apoptosis [60]. Additionally, classical swine fever virus (CSFV) induced autophagy and delayed apoptosis by suppressing the ROS-dependent RLR signaling pathway and facilitating persistent infection in host cells [61].

**Table 1:** Summary of autophagy inducers in cancer cells.

Drugs	Cancer Development	Mechanisms	Cancer Type	Refs
**Natural Products**				
Luteolin	Cell apoptosis	Increased Beclin 1	Liver cancer	[56]
Resveratrol (RSV)	Cell apoptosis	LKB1-AMPK and PI3K/AKT-regulated mTOR signaling pathways	HL-60cell	[62]
Apigenin	Cell apoptosis	Inhibition of PI3K/Akt/mTOR pathway	HCC	[63]
Erastin	Cell ferroptosis	Increased of TfR1expression and lipid ROS accumulation	Hela, lung	[64]
Erianin	Cell ferroptosis	Accumulation of Fe^2+^ and ROS, lipid peroxidation	Colorectal cancer (CRC)	[65]
Ginsenoside Rh4	Cell ferroptosis	Activating ROS/P53 pathway	CRC	[66]
Anomanolide C	Cell ferroptosis	Reduced GPX4 expression, accumulation of Fe^2+^	TNBC	[67]
6-Gingerol	Cell ferroptosis	Increased ROS level and LC3B-II and Beclin-1 expression	Prostate cancer	[68]
Curcumin	Cell ferroptosis	Increased level of Beclin1 and LC3, decreased P62	NSCLC	[69]
Chrysin	Cell ferroptosis	Increased ROS, inhibition of carbonyl reductase1 (CBR1)	PCa	[70]
Urolithin A	Cell metastasis	Decreased the MMP-9 expression	CRC	[71]
Gallotannin (GT)	Cell metastasis	Inhibition of PI3K/Akt/mTOR pathway	CRC	[72]
Halofuginone (HF)	Cell metastasis	Suppression of STMN1 and p53 expression	Breast cancer	[73]
Salidroside	Cell apoptosis	Activation of the AMPK pathway and downregulation of mTOR pathway	HUVECs	[74]
Itraconazole	Cell proliferation	Repression of AKT1-MTOR signaling	Glioblastoma	[75]
Isorhapontigenin (ISO)	Cell growth	MAPK8-JUN-dependent transcriptional induction of SESN2	Bladder cancer	[76]
**Others**				
SGK1	Cell metastasis	Inhibition EMT process	PCa	[77]
Valproic acid (VPA)	Tumor growth	Activation of AMPK and inhibition of downstream MTOR signaling	Lymphoma	[78]

pH-sensitive polymeric nanoparticles loaded with gold(I) compounds were shown to induce autophagy and apoptosis in MCF-7 breast cancer cells by inhibiting thioredoxin reductase, increasing ROS levels, and impairing lysosomes [79]. Plant lectins, of non-immune origin, could block EGFR-mediated survival pathways, leading to autophagic cell death via the modulation of autophagic hub proteins and miRNAs [80]. Resveratrol (RSV), a polyphenol phytoalexin found in grapes and blueberries, induced autophagy-mediated apoptotic cell death in HL-60 cells via the LKB1-AMPK and PI3K/AKT-mTOR signaling pathways [62]. Nimbolide, a terpenoid lactone from neem, induced autophagy-mediated apoptotic cell death in breast cancer cells by modulating epigenetic modifications and regulating Beclin 1, LC3B, p62, and mTOR protein expression [81]. Apigenin, a natural flavonoid found in various fruits and vegetables, triggered apoptosis and autophagy in hepatocellular carcinoma cells by inhibiting the PI3K/Akt/mTOR pathway [63].

#### 3.1.2. Autophagy Inhibitors Regulate Cancer Cell Apoptosis

Oridonin, a diterpenoid from the Chinese herb *Rabdosia rubescens*, Lamiaceae family, induced cell apoptosis in breast cancer cells by suppressing autophagy [82]. In an inflammatory microenvironment, autophagy promoted the apoptosis of mesenchymal stem cells (MSCs) by inhibiting the expression of the pro-survival gene Bcl-2 via the suppression of the ROS/mitogen-activated protein kinase 1/3 pathway [83]. Salinomycin, a monocarboxylic ionophore, could induce breast cancer cell apoptosis and ROS production by blocking autophagy [84]. S-adenosyl-L-methionine (AdoMet), a naturally occurring sulfonium compound, inhibited the proliferation of breast cancer cells MCF-7 by inducing both autophagy and apoptosis. Combining AdoMet with the autophagy inhibitor chloroquine (CLC) synergistically inhibited autophagy, leading to growth inhibition and apoptosis in breast cancer cells [85].

**Table 2:** Summary of autophagy inhibitors in cancer cells.

Drugs	Cancer Development	Mechanisms	Cancer Type	Refs
**Natural Products**				
Oridonin	Cell apoptosis	Inhibition of Beclin 1	Breast cancer	[82]
Salinomycin	Cell apoptosis	ROS production led to mitochondrial dysfunction	Breast cancer	[84]
AdoMet	Cell apoptosis	Enhanced beclin-1 and LC3B-II	Breast cancer	[85]
Metformin	Cell ferroptosis	Inhibiting lncRNA H19, increasing lipid ROS	Breast cancer	[86]
Melatonin + erastin	Cell ferroptosis	Increased lipid ROS and P62	Squamous cell carcinoma	[87]
Deoxypodophyllotoxin (DPT)	Tumor growth	Triggered mitochondrial ROS	PCa	[88]
**miRNAs**				
MIR152	Cell apoptosis	Downregulation of ATG14	Ovarian cancer	[89]
MiR-221	Cell apoptosis	Downregulation of TP53INP1	CRC	[90]
MiRNA-30a	Cell metastasis	Increased beclin-1 and Atg5	HCC	[91]
**Others**				
PCBP1	Cell apoptosis	Downregulation of LC3B	CRC	[92]
IBP	Cell metastasis	Repression of MTORC2 signaling	Breast cancer	[93]
KPNA2	Cell metastasis	Inhibition of p53	OSCC	[94]
MCT4	Cell ferroptosis	Increasing lipid ROS, inhibiting AMPK pathways	Bladder cancer	[95]
H2S	Cell proliferation and differentiation	Elevated LC-3II, ATG5, and Beclin-1	HaCaT cells	[96]

MIR152, a miRNA involved in autophagy regulation, enhances cisplatin-induced apoptosis and inhibits cell proliferation in cisplatin-resistant ovarian cancer by reducing cisplatin-induced autophagy [89]. Poly C binding protein (PCBP1), a tumor suppressor, promotes tumor cell apoptosis during starvation by downregulating LC3B and repressing autophagy. PCBP1 overexpression triggers caspase 3- and 8-mediated apoptosis and downregulates anti-apoptotic Bcl-2 expression. The effect of PCBP1 is synergized via an autophagic inhibitor, indicating its ability to inhibit autophagy and induce apoptosis [92]. FOXO3a, an autophagy-regulating transcription factor, undergoes basal autophagy-mediated turnover, creating a feedback loop. The inhibition of autophagy via FOXO3a stimulates the transcription of the pro-apoptotic BBC3/PUMA gene, sensitizing cells to apoptosis. Autophagy inhibitors can transform the action of MDM2-targeted drugs from growth inhibition to apoptosis, reducing tumor burden [57]. MiR-221, known to modulate proliferation, apoptosis, cell cycle distribution, and cell migration in various cancers, has also been found to regulate autophagy. In colorectal cancer (CRC), miR-221 promotes cell proliferation by inhibiting autophagy and targeting tumor protein 53-induced nuclear protein 1 (TP53INP1) [90].

### 3.2. Interaction between Autophagy and Ferroptosis

Ferroptosis, on the other hand, is a recently discovered form of regulated cell death characterized via lethal lipid ROS accumulation, leading to cell membrane destruction [97]. It is now appreciated as likely one of the most widespread and ancient forms of cell death; unlike apoptosis or necrosis, ferroptosis relies on iron and lipid metabolism. It is triggered via intracellular antioxidant depletion or changes in key lipid metabolism enzymes [98]. Ferroptosis has emerged as a potential therapeutic approach in cancer for selectively targeting cancer cells, especially those with altered iron metabolism or impaired antioxidant defenses [99]. Recent studies have unveiled the intricate crosstalk between autophagy and ferroptosis, the pharmacological modulation of ferroptosis, via both its induction and inhibition, holds great potential for the treatment of various diseases, like cancers [100].

#### 3.2.1. Autophagy Inducers Regulate Cancer Cell Ferroptosis

Efforts are underway to develop cancer therapies that induce ferroptosis. While various nanoparticle-based strategies have been explored to deliver iron, peroxides, and other toxic substances for indiscriminate tumor cell killing, targeted approaches are being developed to exploit the multiple enzymes involved in ferroptosis regulation. Among these enzymes, GPX4 stands out as a potential target due to its expression in numerous cancer cell lines and its crucial role in their survival [100,101]. Anomanolide C, a withanolide isolated from *Tubocapsicum anomalum* (Solanaceae family), suppresses the tumor progression and metastasis in TNBC cells by inducing autophagy-dependent ferroptosis, which leads to Fe^2+^ accumulation via ubiquitinating GPX4 [67].

Erastin induces autophagy, which leads to iron-dependent ferroptosis by degrading ferritin and upregulating transferrin receptor 1 (TfR1) expression [64,102]. Erianin, derived from *Dendrobium nobile Lindl* (Orchidaceae family), inhibits growth and metastasis in KRAS^G13D^ colorectal cancer via autophagy-induced ferroptosisa [65]. Furthermore, another compound ginsenoside Rh4 could also inhibit colorectal cancer cell proliferation by activating autophagy-induced ferroptosis [66]. 6-Gingerol is a bioactive compound isolated from *Zingiber officinale*. Studies found that 6-Gingerol may induce protective autophagy, autophagic cell death, and ferroptosis-mediated cell death in prostate cancer cells [68]. Curcumin is a yellow polyphenol compound derived from the turmeric plant. It could induce ferroptosis via activating autophagy in non-small cell lung cancer (NSCLC) A549 and H1299 cells [69]. Chrysin is a natural bioflavonoid widely found in propolis, honey, and blue passion flowers (*Passiflora caerulea*). Research showed that chrysin enhanced PC sensitivity to gemcitabine by inducing ROS-dependent autophagy-mediated ferroptotic death [70].

#### 3.2.2. Autophagy Inhibitors Regulate Cancer Cell Ferroptosis

Metformin, commonly used for T2D therapy, can induce ferroptosis in breast cancer by inhibiting autophagy via lncRNA-H19 [86]. MCT4, a lactate/proton monocarboxylate transporter 4, is upregulated in patients with bladder cancer and associated with poor prognosis. The knockdown of MCT4 promotes oxidative stress, induces ferroptosis, and inhibits autophagy in bladder cancer [95]. Combined treatment of melatonin and erastin markedly reduces the tumor size in vivo, enhances apoptosis and ferroptosis, and decreases autophagy levels without any systemic side effects [87]. Combining ferroptosis induction-based treatment with other therapeutic approaches, such as immune checkpoint blockade and radiotherapy, could be considered as potential options. Research shows that anti-PDL1 therapy has been shown to enhance the effectiveness of ferroptosis-inducing therapy. Researchers discovered that anti-PDL1 antibodies can stimulate CD8+ T cells to release IFNg, which, in turn, downregulates both subunits of system x_c_− in tumor cells. This downregulation sensitizes cancer cells to ferroptosis, resulting in a synergistic anticancer effect. Combining immunotherapy with ferroptosis induction shows promise as a treatment approach where the two modalities mutually enhance each other [100,103].

The interplay between autophagy and ferroptosis in cancer is a fascinating and evolving field of research; whether a given cancer is more sensitive or resistant to ferroptosis induction is dictated by its specific genetic background [100]. Further investigations are needed to elucidate the molecular mechanisms governing their interdependencies and develop effective therapeutic strategies targeting both autophagy and ferroptosis in cancer treatment. Understanding and harnessing this interplay may exhibit great potential for improving cancer therapies and patient outcomes in the future.

### 3.3. Autophagy Regulates Cancer Cell Metastasis

Autophagy is a catabolic process involved in protein degradation, cell growth regulation, and maintaining cellular homeostasis. It has a significant impact on cancer development, metastasis, and cellular phenotypes, with both pro-tumorigenic and tumor-suppressive roles [104,105].

#### 3.3.1. Autophagy Inducers Regulate Cancer Cell Metastasis

Long non-coding RNA MALAT1 is upregulated in pancreatic cancer and promotes cancer proliferation and metastasis by stimulating autophagy. It interacts with the RNA-binding protein HuR to enhance the post-transcriptional regulation of TIA-1, affecting the autophagic process [105]. Urolithin A, a polyphenol metabolite, induces autophagy in CRC cells. It inhibits cell migration and reduces matrix metalloproteinas-9 (MMP-9) activity. The inhibition of autophagy or caspases suppresses urolithin A-induced cell death and anti-metastatic activity [71]. Serum- and glucocorticoid-induced protein kinase 1 (SGK1) is associated with prostate cancer (PCa) progression and metastasis. SGK1 inhibition attenuates epithelial–mesenchymal transition (EMT) and metastasis, while its overexpression promotes the invasion and migration of PCa cells. The inhibition of SGK1 induces anti-metastatic effects with the autophagy-mediated repression of EMT via the downregulation of Snail. Combined mTOR and SGK1 inhibition enhances autophagy and exhibits synergistic anti-metastatic effects in PCa cells [77].

Gallotannin (GT), a polyphenolic compound, has been shown to suppress the lung metastasis of metastatic CRC cells by inducing apoptosis and autophagy [72]. Cholesterol in lipid rafts plays a crucial role in cancer cell survival during metastasis. Methyl-β-cyclodextrin (MβCD), a polysaccharide that depletes membrane cholesterol, selectively induces cell death in cancer cells. Cholesterol depletion leads to the downregulation of caspase-8 mRNA, indirectly promoting the induction of autophagy. Membrane cholesterol depletion also reduces the migratory efficiency of breast cancer MDA-MB 231 cells [106]. Halofuginone (HF), an analog of quinazolinone alkaloid, inhibits the growth, migration, and invasion of MCF-7 cells. It activates autophagy by suppressing the expressions of STMN1 and p53 [73].

#### 3.3.2. Autophagy Inhibitors Regulate Cancer Cell Metastasis

Autophagy and autophagy-related genes (Atg) have significant roles in tumorigenesis and tumor metastasis [107]. Cadherin-6, a type 2 cadherin, is involved in EMT during embryonic development and is aberrantly re-activated in cancer. In thyroid cancer, cadherin-6 promotes EMT and metastasis by inhibiting autophagy [108]. Interferon regulatory factor-4 binding protein (IBP) is a novel activator of Rho GTPases. Increased expressions of IBP are associated with malignant behaviors in human breast cancer cells, and its expression negatively correlates with cell autophagy. Studies have shown that IBP-mediated breast cancer cell growth and metastasis in vitro and in vivo are strongly linked to the suppression of mTORC2-dependent autophagy. In particular, the inhibition of autophagy occurs via the activation of the mTORC2/Akt/FOXO3a signaling pathway, leading to the increased phosphorylation of Akt on ser473 and FOXO3a on Thr32 [93]. Autophagy is an important survival mechanism under conditions of cell stress, and its inhibition can suppress pulmonary metastasis of hepatocellular carcinoma (HCC) in vivo and in vitro by impairing the anoikis resistance and lung colonization of HCC cells [59].

Hypoxia-regulated autophagy has been found to suppress metastasis in breast cancer by preventing tumor fibrosis. The inhibition of autophagy via the hypoxia-induced expression of the kinase-dead ULK1 mutant K46N increased lung metastases in MDA-MB-231 xenograft mouse models [109]. MiRNA-30a has been shown to inhibit the metastasis of hepatocellular carcinoma in a well-established nude mouse model of lung metastasis via suppressing autophagy-related protein Beclin 1 and ATG5 directly [91]. SIRT1, a NAD+-dependent protein deacetylase that belongs to the mammalian sirtuin family, could facilitate melanoma metastasis by accelerating E-cadherin degradation via inhibiting autophagy via the deacetylation of Beclin 1 [104]. Karyopherin α2 (KPNA2), a member of the importin α family involved in nucleocytoplasmic transport, plays a crucial role in autophagy regulation. The knockdown of KPNA2 inhibits autophagy, suppresses cell migration, and reduces cisplatin resistance in oral aquamous cell carcinoma (OSCC) cell lines, such as CAL-27, SCC-15, and Tca8113. The function of KPNA2 in autophagy is p53-dependent, and by regulating the translocation of p53, KPNA2 induces autophagy to promote the chemoresistance and metastasis of OSCC cells [94].

### 3.4. Autophagy and Cancer Cell Cycle

Autophagy is a process by which cells recycle their own components to maintain cellular homeostasis. It is also known as a form of programmed cell death. [110]. The cell cycle, on the other hand, is a series of events that occur in a cell, leading to its division and duplication of its DNA (DNA replication) to produce two daughter cells.

The cell cycle consists of four phases: G1 phase, S phase (synthesis), G2 phase (collectively known as interphase), and M phase (mitosis). The cell cycle is crucial for the development of a single-cell fertilized egg into a mature organism, as well as for the renewal of tissues such as hair, skin, blood cells, and internal organs. Autophagy and cell cycle arrest have a tight biological relationship. There is a close biological relationship between autophagy and cell cycle arrest. Researchers have used pharmacological methods to induce cell cycle arrest and have found that it is closely linked to autophagy [111]. Previous studies have suggested that autophagy can be activated by increasing the expression of p21, a protein involved in cell cycle regulation [112]. The overexpression of p21 can induce autophagy in breast cancer cells and metabolically inhibit tumor growth [111], indicating that p21 may act as a molecular bridge between autophagy and cell cycle arrest [113]. Studies have also confirmed that autophagy is tied to the process of the cell cycle. For example, the knock out or suppression of the p53 gene, a well-known tumor suppressor gene, has been shown to cause a periodic peak of autophagy around the G1/S phase. Additionally, the neutralization of p53 leads to the suppression of an autophagic program but only during discrete phases of the cell cycle [114].

The cell cycle is tightly controlled by precise mechanisms, and some autophagy genes have been found to contribute to cell cycle progression [115]. ATG5, a critical host-defense mechanism, prevents renal fibrosis by modulating G2/M arrest in proximal epithelial cells and its subsequent effect on COL1 (collagen, type I) production. The renal protection effect of ATG5 is dependent on its autophagic activity [115].

### 3.5. Autophagy in Cancer Proliferation and Differentiation

Regarding the instrumental role of autophagy in cancer development, as mentioned above, such as cell apoptosis, metastasis, and cell cycle, autophagy may also be regulated in other progressions of cancer, such as proliferation and differentiation.

One study demonstrated that hydrogen sulfide (H_2_S) can promote the proliferation and differentiation of keratinocyte (HaCaT) by activating autophagy. Human keratinocyte cells exposed to sodium hydrosulfide (NaHS) as a H_2_S donor showed increased expressions of autophagy-related proteins LC-3II, ATG5, and Beclin-1 in a dose-dependent manner. ATG5 is an essential protein involved in the formation of autophagosomes. When autophagy was blocked using ATG5 siRNA, cell proliferation and differentiation induced via sodium hydrosulfide were inhibited. These findings provide additional insights into the role of autophagy in keratinocyte proliferation and differentiation [96]. Another study focused on a long noncoding RNA called HOTAIRM1, which is associated with myeloid cell differentiation and the degradation of the oncoprotein PML-RARA. It was found that HOTAIRM1 regulates autophagy, and when its expression was reduced, autophagosome formation was inhibited. This suggests that HOTAIRM1 enhances the autophagy pathway, leading to the degradation of the PML-RARA oncoprotein and promoting myeloid cell differentiation [116]. The expression level of the autophagy-related protein Beclin-1 is also relevant to cancer differentiation. A low expression of Beclin-1 has been positively correlated with poor differentiation, lymph node metastasis, distant metastasis, epithelial–mesenchymal transition (EMT) stage, tumor recurrence, and overall patient survival time [117]. These findings suggest that the dysregulation of autophagy, as indicated by the decreased Beclin-1 expression, can contribute to aggressive cancer behavior and poor patient outcomes.

### 3.6. Defective Autophagy in Tumorigenesis

Defective autophagy, characterized by impaired or dysregulated autophagic processes, has been implicated in various aspects of tumorigenesis [118]. When autophagy malfunctions, it can lead to the accumulation of damaged proteins and organelles, genomic instability, and altered cellular metabolism, all of which contribute to tumorigenesis [118,119,120,121]. Understanding the role of defective autophagy in tumorigenesis has prompted research into targeting autophagy as a potential therapeutic strategy. Here, we shortly summarized that defective autophagy impacts different aspects related to tumorigenesis.

Autophagy is intricately connected to cellular metabolism as it supplies recycled components for energy production and biosynthesis [122]. In the context of tumors, impaired autophagy can disrupt metabolic pathways, hinder nutrient recycling, and encourage cancer cells to rely on alternative energy sources, such as aerobic glycolysis (the Warburg effect) [123]. This metabolic reprogramming bestows a growth advantage on cancer cells, facilitating their survival and uncontrolled proliferation [124]. In addition to its role in metabolism, autophagy also has a role in inflammatory and immune responses. It regulated inflammation by removing damaged organelles and protein aggregates [125]. When autophagy becomes defective, it hampers the removal of damaged cellular components, resulting in their accumulation. This accumulation triggers chronic inflammation, which can contribute to the development of tumors [125,126]. Persistent inflammation fosters an environment that promotes cell proliferation, genomic instability, and angiogenesis, all of which support tumor growth and progression [48]. Moreover, autophagy also exhibits a dual role in immunotherapy [127]. On one hand, it enhances the immunogenicity of tumor cells by presenting tumor antigens to immune cells, thereby facilitating tumor recognition and triggering an immune response. On the other hand, defective autophagy can impair immune cell function, specifically in antigen presentation and T-cell activation. Consequently, defective autophagy in tumor cells can limit the effectiveness of immunotherapeutic approaches like immune checkpoint inhibitors or adoptive cell therapies. Further research is needed to elucidate the precise mechanisms and develop therapeutic strategies to manipulate autophagy for cancer treatment.

In addition, research has shown that sphingolipid is a bioactive lipid that regulates various cellular functions, including proliferation, migration, senescence, and cell death, and targeting sphingolipid signaling pathways has emerged as a potential therapeutic strategy. Dysregulation of sphingolipid metabolism can impact the autophagy of mitochondria, or mitophagy, contributing to the development of various diseases, including cancer and neurodegenerative disorders. Modulating sphingolipid levels and their downstream signaling pathways could potentially enhance mitophagy and promote the selective elimination of cancer cells that rely on mitochondrial metabolism for survival and growth. Hence, understanding the role of sphingolipids in mitophagy regulation may have implications for developing novel therapeutic approaches for these diseases as well [128].

## 4. Autophagy in Cancer Therapeutics

Autophagy’s role in tumor biology is complex, with both positive and negative effects on cancer cells. Scientific studies have demonstrated that inhibiting autophagy can enhance the efficacy of anticancer treatments, whereas promoting autophagy can induce cell death under certain conditions. Depending on the inducing factors, duration, and cell type, excessive or sustained autophagy can lead to autophagic tumor death [129,130].

### 4.1. Promotion of Autophagy in Cancer Therapeutics

Chitosan nanoparticles (CS-NPs) have been extensively investigated as potential carriers for drug delivery in cancer treatment. They have also been identified as a novel autophagy initiator at nontoxic concentrations ranging from 10 to 100 μg/mL. CS-NPs have been found to induce autophagy and regulate cellular apoptosis. The induction of autophagy by CS-NPs is characterized by an increase in the ratio of LC3 II to LC3 I, and it has been shown that CS-NPs-mediated autophagy is associated with the generation of ROS. When the ROS scavenger N-acetylcysteine is used, it attenuates the CS-NPs-induced autophagy. These findings suggest that CS-NPs are capable of inducing protective autophagy via ROS generation, which inhibits tumor cell death [131].

Salidroside, which is derived from the traditional Chinese medicine *Rhodiola crenulata* (Crassulaceae family), has been found to increase autophagy and reduce apoptosis in human umbilical vein endothelial cells (HUVECs) under oxidative stress. This effect is dose-dependent and is achieved via the activation of the AMPK pathway and the downregulation of the mTOR pathway [74]. The histone deacetylase (HDAC) inhibitor valproic acid (VPA) synergistically interacts with chemotherapeutic agents to induce autophagy in lymphoma cells and enhance their sensitivity to chemotherapy. This is accomplished by activating AMPK and inhibiting downstream MTOR signaling [78].

Itraconazole, a traditional antifungal drug, has emerged as a potential anti-cancer agent capable of inhibiting the proliferation of glioblastoma cells both in vitro and in vivo via the induction of autophagy via the repression of AKT1-MTOR signaling [75]. Isorhapontigenin (ISO), a derivative of stilbene found in the Chinese herb *Gnetum cleistostachyum* (Gnetaceae family), has been shown to induce autophagy and inhibit the growth of bladder cancer. This effect is mediated via the MAPK8-JUN pathway, which transcriptionally induces SESN2 [76]. Gefitinib (GEF), an inhibitor of EGFR tyrosine kinase, has been found to induce autophagy in NSCLC cell lines, and the combined treatment with clarithromycin (CAM), a macrolide antibiotic having the effect of inhibiting autophagy flux, enhances the cytotoxic effect in NSCLC cell lines. These results suggest that GEF-induced autophagy has a cytoprotective function and indicates the therapeutic potential of using CAM in GEF therapy [132].

### 4.2. Inhibition of Autophagy in Cancer Therapeutics

Deoxypodophyllotoxin (DPT), a naturally occurring flavolignan isolated from *Anthriscus sylvestris* (Apiaceae family), has demonstrated the ability to inhibit tumor growth in PCa via suppression of autophagy by triggering mitochondrial ROS in vivo and in vitro [88]. cAMP response element-binding protein 1 (CREB1), a nuclear transcription factor, is involved in the regulation of genes associated with cell survival and death. In autophagy-defective cells, CREB1 induces DNA damage and subsequent apoptosis in response to etoposide. Reactivating CREB1 or inhibiting autophagy not only enhances the efficacy of rapamycin but also mitigates the chemoresistance mediated by MTOR inhibition [133]. The combination of temsirolimus (TEM), an MTOR inhibitor, and hydroxychloroquine (HCQ), an autophagy inhibitor, has been found to enhance cell death in preclinical models. This study suggests that the combination of TEM and HCQ modulates autophagy in patients and exhibits significant antitumor activity [134].

Salinomycin, a monocarboxylic ionophore, induces apoptosis and the production of ROS, both of which are blocked by autophagy, resulting in the protection of cancer cells. This interplay between autophagy and apoptosis induced via salinomycin sheds light on the relationship between these two physiological responses in cancer cells [84]. Acid-sensing ion channels (ASICs), which are voltage-insensitive cation channels in the epithelial Na+ channel/degenerin superfamily, have been implicated in various physiological and pathological conditions. Studies have demonstrated that the downregulation of ASICs inhibits gastric cancer growth by reducing autophagy [135]. These studies highlight the feasibility of inhibiting autophagy to promote apoptosis and suggest that combining autophagy inhibitors with anti-tumor therapies may lead to more effective cancer treatment.

The above studies have shown that excessive ROS production, resulting from cellular metabolism, can lead to oxidative stress and damage various cellular components, including proteins, lipids, and DNA, which contributes to the development of diseases including cancer [136]. Ceramides, the base of all sphingolipids, are formed when a fatty acid binds to the amino group. Some ceramides have been associated with mitochondrial dysfunction, including suppressing the mitochondrial respiratory chain, increasing ROS levels, and reducing mitochondrial membrane potential [137]. Autophagy, the process of degrading and recycling cellular components, helps the cells cope with oxidative stress, eliminates ROS, and maintains cellular redox balance. By selectively removing damaged mitochondria, proteins, and organelles, autophagy prevents ROS accumulation and protects against tumor development [138]. However, the relationship between autophagy and ROS is complex, and the effects may vary depending on the cellular context [139]. Further research is needed to fully understand the interplay between autophagy, ROS, and tumorigenesis, thus uncovering new therapeutical targets for cancer therapy.

## 5. Conclusions and Outlooks

Autophagy plays a crucial role in regulating various aspects of tumor cell homeostasis, including the occurrence and development of cell apoptosis, metastasis, and cell cycle arrest [140]. Throughout this review, it has become evident that interventions that activate or inhibit autophagy hold significant therapeutic potential. As a tumor suppressor mechanism, autophagy can induce tumor cell death, prevent tumor formation, and decrease the probability of DNA mutation [141]. This autophagy-mediated cell death is often referred to as “autophagic cell death” or “autophagic programmed cell death”, characterized by the excessive activation of autophagy leading to uncontrolled self-degradation and cell death [142]. However, the classification of autophagy as a type of cell death is still a topic of ongoing research and debate. Autophagy can interact with other forms of cell death, such as apoptosis or ferroptosis, and the specific outcomes may depend on the context and cellular conditions. Autophagy also serves as a tumor protective mechanism, enabling tumor cells to withstand nutrient deficiency, radiotherapy, chemotherapy, and other harsh conditions, thereby helping them escape apoptosis and promoting cell survival [143].

Dysregulated autophagy has been implicated in the development of various types of cancer [144]. With advancements in gene and molecular technology, our understanding of autophagy will become more comprehensive and profound. A deeper understanding of the complex interplay between autophagy and other cellular processes, such as apoptosis and immune response will facilitate a better explanation of its dual role in cancer development and progression. It may also pave the way for autophagy to emerge as a new target for cancer therapy. By unraveling the mechanisms underlying autophagy, novel anti-cancer therapeutics can be developed, revolutionizing the field of cancer treatment. However, further research is necessary to fully harness the benefits of targeting autophagy in cancer treatment.

## Data Availability

Not applicable.

## Abbreviations

AdoMet	S-adenosyl-L-methionine
AL	autophagolysosome
ATG	autophagy related
Atg	autophagy-related genes
ASICs	acid-sensing ion channels
CAM	clarithromycin
CBR1	carbonyl reductase1
CLC	chloroquine
CMA	chaperone-mediated autophagy
CRC	colorectal cancer
CSFV	classical swine fever virus
CS-NPs	chitosan nanoparticles
CREB1	cAMP response element-binding protein 1
DPT	deoxypodophyllotoxin
GEF	gefitinib
EMT	epithelial–mesenchymal transition
GQ	7-O-geranylquercertin
GT	gallotannin
HaCaT	human keratinocytes
HCC	hepatocellular carcinoma
HCQ	hydroxychloroquine
HDAC	histone deacetylase
HF	halofuginone
H2S	hydrogen sulfide
HUVECs	human umbilical vein endothelial cells
IBP	interferon regulatory factor-4 binding protein
ISO	isorhapontigenin
KPNA2	karyopherin α2
LC3B	microtubule-associated protein light chain 3B (LC3B)
MSCs	mesenchymal stem cells
mTOR	mammalian target of rapamycin
MβCD	methyl-β-cyclodextrin
MM9	matrix metalloproteinas-9
NSCLC	non-small cell lung cancer
OSCC	oral squamous cell carcinoma
PE	phosphatidylethanolamine
PCBP1	poly C binding protein
PI3K	phosphoinositide 3-kinase
PCa	prostate cancer
RSV	resveratrol
ROS	reactive oxygen species
SGK1	serum- and glucocorticoid-induced protein kinase 1
TEM	temsirolimus
TP53INP1	tumor protein 53-induced nuclear protein 1
Ubl	ubiquitin-like
ULK1	unc-51 like autophagy activating kinase
VPA	valproic acid

## References

[1] Safaralizadeh R., Beilankouhi E.A.V., Valilo M., Dastmalchi N., Teimourian S. (2023). The function of autophagy in the initiation, and development of breast cancer. Curr. Med. Chem..

[2] Debnath J., Gammoh N., Ryan K.M. (2023). Autophagy and autophagy-related pathways in cancer. Nat. Rev. Mol. Cell Biol..

[3] Amaravadi R., Kimmelman A.C., White E. (2016). Recent insights into the function of autophagy in cancer. Genes Dev..

[4] Laplante M., Sabatini D.M. (2012). mTOR Signaling in Growth Control and Disease. Cell.

[5] Levy J.M.M., Thorburn A. (2011). Targeting autophagy during cancer therapy to improve clinical outcomes. Pharmacol. Ther..

[6] Levy J.M.M., Towers C.G., Thorburn A. (2017). Targeting autophagy in cancer. Nat. Rev. Cancer.

[7] Li W.-W., Li J., Bao J.-K. (2011). Microautophagy: Lesser-known self-eating. Cell. Mol. Life Sci..

[8] Ghosh R., Pattison J.S. (2018). Macroautophagy and Chaperone-Mediated Autophagy in Heart Failure: The Known and the Unknown. Oxidative Med. Cell. Longev..

[9] Yamamoto H., Matsui T. (2023). Molecular mechanisms of macroautophagy, microautophagy, and chaperone-mediated autophagy. J. Nippon. Med. Sch..

[10] Cheon S.Y., Kim H., Rubinsztein D.C., Lee J.E. (2019). Autophagy, Cellular Aging and Age-related Human Diseases. Exp. Neurobiol..

[11] Klionsky D.J., Petroni G., Amaravadi R.K., Baehrecke E.H., Ballabio A., Boya P., Pedro J.M.B., Cadwell K., Cecconi F., Choi A.M.K. (2021). Autophagy in major human diseases. EMBO J..

[12] Boellaard J.W., Schlote W., Tateishi J. (1989). Neuronal autophagy in experimental Creutzfeldt-Jakob’s disease. Acta Neuropathol..

[13] Zhang H., Mahuran D.J., Callahan J.W. (2010). Identification of proteins in the ceroid-like autofluorescent aggregates from liver lysosomes of Beige, a mouse model for human Chediak–Higashi syndrome. Mol. Genet. Metab..

[14] Nixon R.A. (2007). Autophagy, amyloidogenesis and Alzheimer disease. J. Cell Sci..

[15] Arias E., Cuervo A.M. (2011). Chaperone-mediated autophagy in protein quality control. Curr. Opin. Cell Biol..

[16] Yang Q., She H., Gearing M., Colla E., Lee M., Shacka J.J., Mao Z. (2009). Regulation of Neuronal Survival Factor MEF2D by Chaperone-Mediated Autophagy. Science.

[17] Zhou D., Li P., Lin Y., Lott J.M., Hislop A.D., Canaday D.H., Brutkiewicz R.R., Blum J.S. (2005). Lamp-2a Facilitates MHC Class II Presentation of Cytoplasmic Antigens. Immunity.

[18] Sooparb S., Price S.R., Shaoguang J., Franch H.A. (2004). Suppression of chaperone-mediated autophagy in the renal cortex during acute diabetes mellitus. Kidney Int..

[19] Nair U., Klionsky D.J. (2005). Molecular Mechanisms and Regulation of Specific and Nonspecific Autophagy Pathways in Yeast. J. Biol. Chem..

[20] Klionsky D.J., Cregg J.M., Dunn W.A., Emr S.D., Sakai Y., Sandoval I.V., Sibirny A., Subramani S., Thumm M., Veenhuis M. (2003). A Unified Nomenclature for Yeast Autophagy-Related Genes. Dev. Cell.

[21] Nakatogawa H., Suzuki K., Kamada Y., Ohsumi Y. (2009). Dynamics and diversity in autophagy mechanisms: Lessons from yeast. Nat. Rev. Mol. Cell Biol..

[22] Funderburk S.F., Wang Q.J., Yue Z. (2010). The Beclin 1-VPS34 complex--at the crossroads of autophagy and beyond. Trends Cell. Biol..

[23] Azad M.B., Chen Y., Henson E.S., Cizeau J., McMillan-Ward E., Israels S.J., Gibson S.B. (2008). Hypoxia induces autophagic cell death in apoptosis-competent cells through a mechanism involving BNIP3. Autophagy.

[24] Chen Y., McMillan-Ward E., Kong J., Israels S., Gibson S.B. (2007). Oxidative stress induces autophagic cell death independent of apoptosis in transformed and cancer cells. Cell Death Differ..

[25] Gutierrez M.G., Master S.S., Singh S.B., Taylor G.A., Colombo M.I., Deretic V. (2004). Autophagy is a defense mechanism inhibiting BCG and Mycobacterium tuberculosis survival in infected macrophages. Cell.

[26] Itakura E., Kishi C., Inoue K., Mizushima N., Hegedűs K., Takáts S., Boda A., Jipa A., Nagy P., Varga K. (2008). Beclin 1 Forms Two Distinct Phosphatidylinositol 3-Kinase Complexes with Mammalian Atg14 and UVRAG. Mol. Biol. Cell.

[27] Liang X.H., Jackson S., Seaman M., Brown K., Kempkes B., Hibshoosh H., Levine B. (1999). Induction of autophagy and inhibition of tumorigenesis by beclin 1. Nature.

[28] Takegawa K., Dewald D.B., Emr S.D. (1995). Schizosaccharomyces pombe Vps34p, a phosphatidylinositol-specific PI 3-kinase essential for normal cell growth and vacuole morphology. J. Cell Sci..

[29] Chen Y., Klionsky D.J. (2011). The regulation of autophagy—Unanswered questions. J. Cell Sci..

[30] Hara T., Takamura A., Kishi C., Iemura S.-I., Natsume T., Guan J.-L., Mizushima N. (2008). FIP200, a ULK-interacting protein, is required for autophagosome formation in mammalian cells. J. Cell Biol..

[31] Cuyàs E., Corominas-Faja B., Joven J., Menendez J.A. (2014). Cell Cycle Regulation by the Nutrient-Sensing Mammalian Target of Rapamycin (mTOR) Pathway. Methods in Molecular Biology.

[32] Xu K., Liu P., Wei W. (2014). mTOR signaling in tumorigenesis. Biochim. Biophys. Acta Rev. Cancer.

[33] Yu L., McPhee C.K., Zheng L., Mardones G.A., Rong Y., Peng J., Mi N., Zhao Y., Liu Z., Wan F. (2010). Termination of autophagy and reformation of lysosomes regulated by mTOR. Nature.

[34] Jung C.H., Jun C.B., Ro S.-H., Kim Y.-M., Otto N.M., Cao J., Kundu M., Kim D.-H. (2009). ULK-Atg13-FIP200 Complexes Mediate mTOR Signaling to the Autophagy Machinery. Mol. Biol. Cell.

[35] Suzuki K., Kubota Y., Sekito T., Ohsumi Y. (2007). Hierarchy of Atg proteins in pre-autophagosomal structure organization. Genes Cells.

[36] Mizushima N. (2007). Autophagy: Process and function. Genes Dev..

[37] Wang J., Davis S., Zhu M., Miller E., Ferro-Novick S. (2017). Autophagosome formation: Where the secretory and autophagy pathways meet. Autophagy.

[38] Mizushima N., Komatsu M. (2011). Autophagy: Renovation of Cells and Tissues. Cell.

[39] Tooze S.A., Yoshimori T. (2010). The origin of the autophagosomal membrane. Nature.

[40] Mizushima N., Noda T., Ohsumi Y. (1999). Apg16p is required for the function of the Apg12p–Apg5p conjugate in the yeast autophagy pathway. EMBO J..

[41] Kaur J., Debnath J. (2015). Autophagy at the crossroads of catabolism and anabolism. Nat. Rev. Mol. Cell Biol..

[42] Ichimura Y., Kirisako T., Takao T., Satomi Y., Shimonishi Y., Ishihara N., Mizushima N., Tanida I., Kominami E., Ohsumi M. (2000). A ubiquitin-like system mediates protein lipidation. Nature.

[43] Kirisako T., Ichimura Y., Okada H., Kabeya Y., Mizushima N., Yoshimori T., Ohsumi M., Takao T., Noda T., Ohsumi Y. (2000). The reversible modification regulates the membrane-binding state of Apg8/Aut7 essential for autophagy and the cytoplasm to vacuole targeting pathway. J. Cell Biol..

[44] Mann S.S., Hammarback J.A. (1994). Molecular characterization of light chain 3. A microtubule binding subunit of MAP1A and MAP1B. J. Biol. Chem..

[45] Kirisako T., Baba M., Ishihara N., Miyazawa K., Ohsumi M., Yoshimori T., Noda T., Ohsumi Y. (1999). Formation process of autophagosome is traced with Apg8/Aut7p in yeast. J. Cell Biol..

[46] Yu J.H., Liu C.Y., Zheng G.B., Zhang L.Y., Yan M.H., Zhang W.Y., Meng X.Y., Yu X.F. (2013). Pseudolaric Acid B Induced Cell Cycle Arrest, Autophagy and Senescence in Murine Fibrosarcoma L929 Cell. Int. J. Med. Sci..

[47] Zhou H., Cheang T., Su F., Zheng Y., Chen S., Feng J., Pei Z., Chen L. (2018). Melatonin inhibits rotenone-induced SH-SY5Y cell death via the downregulation of Dynamin-Related Protein 1 expression. Eur. J. Pharmacol..

[48] White E., Karp C., Strohecker A.M., Guo Y., Mathew R. (2010). Role of autophagy in suppression of inflammation and cancer. Curr. Opin. Cell Biol..

[49] Itakura E., Kishi-Itakura C., Mizushima N. (2012). The hairpin-type tail-anchored SNARE syntaxin 17 targets to autophagosomes for fusion with endosomes/lysosomes. Cell.

[50] Wang Y., Li L., Hou C., Lai Y., Long J., Liu J., Zhong Q., Diao J. (2016). SNARE-mediated membrane fusion in autophagy. Semin. Cell Dev. Biol..

[51] Jäger S., Bucci C., Tanida I., Ueno T., Kominami E., Saftig P., Eskelinen E.-L. (2004). Role for Rab7 in maturation of late autophagic vacuoles. J. Cell Sci..

[52] Liang C., Lee J.S., Inn K.S., Gack M.U., Li Q., Roberts E.A., Vergne I., Deretic V., Feng P., Akazawa C. (2008). Beclin1-binding UVRAG targets the class C Vps complex to coordinate autophagosome maturation and endocytic trafficking. Nat. Cell Biol..

[53] Parzych K.R., Klionsky D.J. (2014). An Overview of Autophagy: Morphology, Mechanism, and Regulation. Antioxidants Redox Signal..

[54] He C., Klionsky D.J. (2009). Regulation mechanisms and signaling pathways of autophagy. Annu. Rev. Genet..

[55] Sardiello M., Palmieri M., Di Ronza A., Medina D.L., Valenza M., Gennarino V.A., Di Malta C., Donaudy F., Embrione V., Polishchuk R.S. (2009). A Gene Network Regulating Lysosomal Biogenesis and Function. Science.

[56] Cao Z., Zhang H., Cai X., Fang W., Chai D., Wen Y., Chen H., Chu F., Zhang Y. (2017). Luteolin Promotes Cell Apoptosis by Inducing Autophagy in Hepatocellular Carcinoma. Cell. Physiol. Biochem..

[57] Fitzwalter B.E., Towers C.G., Sullivan K.D., Andrysik Z., Hoh M., Ludwig M., O’Prey J., Ryan K.M., Espinosa J.M., Morgan M.J. (2018). Autophagy Inhibition Mediates Apoptosis Sensitization in Cancer Therapy by Relieving FOXO3a Turnover. Dev. Cell.

[58] Huang Q., Ou Y.S., Tao Y., Yin H., Tu P.H. (2016). Apoptosis and autophagy induced by pyropheophorbide-alpha methyl ester-mediated photodynamic therapy in human osteosarcoma MG-63 cells. Apoptosis.

[59] Peng Y.-F., Shi Y.-H., Ding Z.-B., Ke A.-W., Gu C.-Y., Hui B., Zhou J., Qiu S.-J., Dai Z., Fan J. (2013). Autophagy inhibition suppresses pulmonary metastasis of HCC in mice via impairing anoikis resistance and colonization of HCC cells. Autophagy.

[60] Wang E.-X., Zou B.-Y., Shi L., Du L.-Y., Zhu Y.-Y., Jiang Y.-M., Ma X.-D., Kang X.-H., Wang C.-Y., Zhen Y.-H. (2017). 7-*O*-geranylquercetin-induced autophagy contributes to apoptosis via ROS generation in human non-small cell lung cancer cells. Life Sci..

[61] Pei J., Deng J., Ye Z., Wang J., Gou H., Liu W., Zhao M., Liao M., Yi L., Chen J. (2016). Absence of autophagy promotes apoptosis by modulating the ROS-dependent RLR signaling pathway in classical swine fever virus-infected cells. Autophagy.

[62] Fan Y., Chiu J.-F., Liu J., Deng Y., Xu C., Zhang J., Li G. (2018). Resveratrol induces autophagy-dependent apoptosis in HL-60 cells. BMC Cancer.

[63] Yang J., Pi C., Wang G. (2018). Inhibition of PI3K/Akt/mTOR pathway by apigenin induces apoptosis and autophagy in hepatocellular carcinoma cells. Biomed. Pharmacother..

[64] Shibata Y., Yasui H., Higashikawa K., Miyamoto N., Kuge Y. (2019). Erastin, a ferroptosis-inducing agent, sensitized cancer cells to X-ray irradiation via glutathione starvation in vitro and in vivo. PLoS ONE.

[65] Miao Q., Deng W.Q., Lyu W.Y., Sun Z.T., Fan S.R., Qi M., Qiu S.H., Zhu Y.R., Lin J.P., Chen M.F. (2023). Erianin inhibits the growth and metastasis through autophagy-dependent ferroptosis in KRAS(G13D) colorectal cancer. Free Radic. Biol. Med..

[66] Wu Y., Pi D., Chen Y., Zuo Q., Zhou S., Ouyang M. (2022). Ginsenoside Rh4 Inhibits Colorectal Cancer Cell Proliferation by Inducing Ferroptosis via Autophagy Activation. Evid. Based Complement. Altern. Med..

[67] Chen Y.-M., Xu W., Liu Y., Zhang J.-H., Yang Y.-Y., Wang Z.-W., Sun D.-J., Li H., Liu B., Chen L.-X. (2023). Anomanolide C suppresses tumor progression and metastasis by ubiquitinating GPX4-driven autophagy-dependent ferroptosis in triple negative breast cancer. Int. J. Biol. Sci..

[68] Liu C.M., An L., Wu Z., Ouyang A.J., Su M., Shao Z., Lin Y., Liu X., Jiang Y. (2022). 6-Gingerol suppresses cell viability, migration and invasion via inhibiting EMT, and inducing autophagy and ferroptosis in LPS-stimulated and LPS-unstimulated prostate cancer cells. Oncol. Lett..

[69] Tang X., Ding H., Liang M., Chen X., Yan Y., Wan N., Chen Q., Zhang J., Cao J. (2021). Curcumin induces ferroptosis in non-small-cell lung cancer via activating autophagy. Thorac. Cancer.

[70] Zhou L., Yang C., Zhong W., Wang Q., Zhang D., Zhang J., Xie S., Xu M. (2021). Chrysin induces autophagy-dependent ferroptosis to increase chemosensitivity to gemcitabine by targeting CBR1 in pancreatic cancer cells. Biochem. Pharmacol..

[71] Zhao W., Shi F., Guo Z., Zhao J., Song X., Yang H. (2018). Metabolite of ellagitannins, urolithin A induces autophagy and inhibits metastasis in human sw620 colorectal cancer cells. Mol. Carcinog..

[72] Mun J.G., Han Y.H., Jeon H.D., Yoon D.H., Lee Y.G., Hong S.H., Kee J.Y. (2021). Inhibitory Effect of Gallotannin on Lung Metastasis of Metastatic Colorectal Cancer Cells by Inducing Apoptosis, Cell Cycle Arrest and Autophagy. Am. J. Chin. Med..

[73] Xia X., Wang L., Zhang X., Wang S., Lei L., Cheng L., Xu Y., Sun Y., Hang B., Zhang G. (2018). Halofuginone-induced autophagy suppresses the migration and invasion of MCF-7 cells via regulation of STMN1 and p53. J. Cell. Biochem..

[74] Zheng X.T., Wu Z.H., Wei Y., Dai J.J., Yu G.F., Yuan F., Ye L.C. (2017). Induction of autophagy by salidroside through the AMPK-mTOR pathway protects vascular endothelial cells from oxidative stress-induced apoptosis. Mol. Cell. Biochem..

[75] Liu R., Li J., Zhang T., Zou L., Chen Y., Wang K., Lei Y., Yuan K., Li Y., Lan J. (2014). Itraconazole suppresses the growth of glioblastoma through induction of autophagy: Involvement of abnormal cholesterol trafficking. Autophagy.

[76] Liang Y., Zhu J., Huang H., Xiang D., Li Y., Zhang D., Li J., Wang Y., Jin H., Jiang G. (2016). SESN2/sestrin 2 induction-mediated autophagy and inhibitory effect of isorhapontigenin (ISO) on human bladder cancers. Autophagy.

[77] Liu W., Wang X., Wang Y., Dai Y., Xie Y., Ping Y., Yin B., Yu P., Liu Z., Duan X. (2018). SGK1 inhibition-induced autophagy impairs prostate cancer metastasis by reversing EMT. J. Exp. Clin. Cancer Res..

[78] Ji M.-M., Wang L., Zhan Q., Xue W., Zhao Y., Zhao X., Xu P.-P., Shen Y., Liu H., Janin A. (2015). Induction of autophagy by valproic acid enhanced lymphoma cell chemosensitivity through HDAC-independent and IP3-mediated PRKAA activation. Autophagy.

[79] Lin Y.X., Gao Y.J., Wang Y., Qiao Z.Y., Fan G., Qiao S.L., Zhang R.X., Wang L., Wang H. (2015). pH-Sensitive Polymeric Nanoparticles with Gold(I) Compound Payloads Synergistically Induce Cancer Cell Death through Modulation of Autophagy. Mol. Pharm..

[80] Fu L.L., Zhao X., Xu H.L., Wen X., Wang S.Y., Liu B., Bao J.K., Wei Y.Q. (2012). Identification of microRNA-regulated autophagic pathways in plant lectin-induced cancer cell death. Cell Prolif..

[81] Pooladanda V., Bandi S., Mondi S.R., Gottumukkala K.M., Godugu C. (2018). Nimbolide epigenetically regulates autophagy and apoptosis in breast cancer. Toxicol. Vitr..

[82] Jou Y.J., Chen C.J., Liu Y.C., Way T.D., Lai C.H., Hua C.H., Wang C.Y., Huang S.H., Kao J.Y., Lin C.W. (2015). Quantitative phosphoproteomic analysis reveals gamma-bisabolene inducing p53-mediated apoptosis of human oral squamous cell carcinoma via HDAC2 inhibition and ERK1/2 activation. Proteomics.

[83] Dang S., Yu Z.-M., Zhang C.-Y., Zheng J., Li K.-L., Wu Y., Qian L.-L., Yang Z.-Y., Li X.-R., Zhang Y. (2015). Autophagy promotes apoptosis of mesenchymal stem cells under inflammatory microenvironment. Stem Cell Res. Ther..

[84] Kim K.-Y., Park K.I., Kim S.-H., Yu S.-N., Lee D., Kim Y.W., Noh K.T., Ma J.Y., Seo Y.-K., Ahn S.-C. (2017). Salinomycin Induces Reactive Oxygen Species and Apoptosis in Aggressive Breast Cancer Cells as Mediated with Regulation of Autophagy. Anticancer. Res..

[85] Cave D.D., Desiderio V., Mosca L., Ilisso C.P., Mele L., Caraglia M., Cacciapuoti G., Porcelli M. (2018). S-Adenosylmethionine-mediated apoptosis is potentiated by autophagy inhibition induced by chloroquine in human breast cancer cells. J. Cell. Physiol..

[86] Chen J., Qin C., Zhou Y., Chen Y., Mao M., Yang J. (2021). Metformin may induce ferroptosis by inhibiting autophagy via lncRNA H19 in breast cancer. FEBS Open Bio.

[87] Wang L., Wang C., Li X., Tao Z., Zhu W., Su Y., Choi W.S. (2023). Melatonin and erastin emerge synergistic anti-tumor effects on oral squamous cell carcinoma by inducing apoptosis, ferroptosis, and inhibiting autophagy through promoting ROS. Cell. Mol. Biol. Lett..

[88] Kim S.H., Kim K.Y., Park S.G., Yu S.N., Kim Y.W., Nam H.W., An H.H., Kim Y.W., Ahn S.C. (2017). Mitochondrial ROS activates ERK/autophagy pathway as a protected mechanism against deoxypodophyllotoxin-induced apoptosis. Oncotarget.

[89] He J., Yu J.-J., Xu Q., Wang L., Zheng J.Z., Liu L.-Z., Jiang B.-H. (2015). Downregulation of ATG14 by EGR1-MIR152 sensitizes ovarian cancer cells to cisplatin-induced apoptosis by inhibiting cyto-protective autophagy. Autophagy.

[90] Liao D., Li T., Ye C., Zeng L., Li H., Pu X., Ding C., He Z., Huang G. (2017). miR-221 inhibits autophagy and targets TP53INP1 in colorectal cancer cells. Exp. Ther. Med..

[91] Fu X.-T., Shi Y.-H., Zhou J., Peng Y.-F., Liu W.-R., Shi G.-M., Gao Q., Wang X.-Y., Song K., Fan J. (2018). MicroRNA-30a suppresses autophagy-mediated anoikis resistance and metastasis in hepatocellular carcinoma. Cancer Lett..

[92] Zhang W., Shi H., Zhang M., Liu B., Mao S., Li L., Tong F., Liu G., Yang S., Wang H. (2016). Poly C binding protein 1 represses autophagy through downregulation of LC3B to promote tumor cell apoptosis in starvation. Int. J. Biochem. Cell Biol..

[93] Chen S., Han Q., Wang X., Yang M., Zhang Z., Li P., Chen A., Hu C., Li S. (2013). IBP-mediated suppression of autophagy promotes growth and metastasis of breast cancer cells via activating mTORC2/Akt/FOXO3a signaling pathway. Cell Death Dis..

[94] Lin F., Gao L., Su Z., Cao X., Zhan Y., Li Y., Zhang B. (2018). Knockdown of KPNA2 inhibits autophagy in oral squamous cell carcinoma cell lines by blocking p53 nuclear translocation. Oncol. Rep..

[95] Dong S., Zheng L., Jiang T. (2023). Loss of Lactate/Proton Monocarboxylate Transporter 4 Induces Ferroptosis via the AMPK/ACC Pathway and Inhibition of Autophagy on Human Bladder Cancer 5637 Cell Line. J. Oncol..

[96] Xie X., Dai H., Zhuang B., Chai L., Xie Y., Li Y. (2016). Exogenous hydrogen sulfide promotes cell proliferation and differentiation by modulating autophagy in human keratinocytes. Biochem. Biophys. Res. Commun..

[97] Chen F., Cai X., Kang R., Liu J., Tang D. (2023). Autophagy-Dependent Ferroptosis in Cancer. Antioxid. Redox Signal..

[98] Dixon S.J., Lemberg K.M., Lamprecht M.R., Skouta R., Zaitsev E.M., Gleason C.E., Patel D.N., Bauer A.J., Cantley A.M., Yang W.S. (2012). Ferroptosis: An iron-dependent form of nonapoptotic cell death. Cell.

[99] Zhang C., Liu X., Jin S., Chen Y., Guo R. (2022). Ferroptosis in cancer therapy: A novel approach to reversing drug resistance. Mol. Cancer.

[100] Jiang X., Stockwell B.R., Conrad M. (2021). Ferroptosis: Mechanisms, biology and role in disease. Nat. Rev. Mol. Cell Biol..

[101] Yang W.S., SriRamaratnam R., Welsch M.E., Shimada K., Skouta R., Viswanathan V.S., Cheah J.H., Clemons P.A., Shamji A.F., Clish C.B. (2014). Regulation of Ferroptotic Cancer Cell Death by GPX4. Cell.

[102] Liu L., Li L., Li M., Luo Z. (2021). Autophagy-Dependent Ferroptosis as a Therapeutic Target in Cancer. Chemmedchem.

[103] Wang W., Green M., Choi J.E., Gijón M., Kennedy P.D., Johnson J.K., Liao P., Lang X., Kryczek I., Sell A. (2019). CD8+ T cells regulate tumour ferroptosis during cancer immunotherapy. Nature.

[104] Sun T., Jiao L., Wang Y., Yu Y., Ming L. (2018). SIRT1 induces epithelial-mesenchymal transition by promoting autophagic degradation of E-cadherin in melanoma cells. Cell Death Dis..

[105] Li L., Chen H., Gao Y., Wang Y.-W., Zhang G.-Q., Pan S.-H., Ji L., Kong R., Wang G., Jia Y.-H. (2016). Long Noncoding RNA MALAT1 Promotes Aggressive Pancreatic Cancer Proliferation and Metastasis via the Stimulation of Autophagy. Mol. Cancer Ther..

[106] Kumar M., Irungbam K., Kataria M. (2018). Depletion of membrane cholesterol compromised caspase-8 imparts in autophagy induction and inhibition of cell migration in cancer cells. Cancer Cell Int..

[107] Guo H., Chitiprolu M., Roncevic L., Javalet C., Hemming F.J., Trung M.T., Meng L., Latreille E., de Souza C.T., McCulloch D. (2017). Atg5 Disassociates the VV-ATPase to Promote Exosome Production and Tumor Metastasis Independent of Canonical Macroautophagy. Dev. Cell.

[108] Gugnoni M., Sancisi V., Gandolfi G., Manzotti G., Ragazzi M., Giordano D., Tamagnini I., Tigano M., Frasoldati A., Piana S. (2016). Cadherin-6 promotes EMT and cancer metastasis by restraining autophagy. Oncogene.

[109] Dower C.M., Bhat N., Wang E.W., Wang H.G. (2017). Selective Reversible Inhibition of Autophagy in Hypoxic Breast Cancer Cells Promotes Pulmonary Metastasis. Cancer Res..

[110] Han Z.-B., Zhang P., Fu Q., Li X.-L., Ge J.-N., Tao D.-D., Hu J.-B., Gong J.-P. (2006). Inducement of tumor cell autophagy and cell cycle analysis. Ai Zheng.

[111] Capparelli C., Chiavarina B., Whitaker-Menezes D., Pestell T.G., Pestell R.G., Hulit J., Andò S., Howell A., Martinez-Outschoorn U.E., Sotgia F. (2012). CDK inhibitors (p16/p19/p21) induce senescence and autophagy in cancer-associated fibroblasts, “fueling” tumor growth via paracrine interactions, without an increase in neo-angiogenesis. Cell Cycle.

[112] Li X., Wang J., Ye Z., Li J.-C. (2012). Oridonin Up-regulates Expression of *P21* and Induces Autophagy and Apoptosis in Human Prostate Cancer Cells. Int. J. Biol. Sci..

[113] Chen K., Shou L.M., Lin F., Duan W.M., Wu M.Y., Xie X., Xie Y.F., Li W., Tao M. (2014). Artesunate induces G2/M cell cycle arrest through autophagy induction in breast cancer cells. Anticancer Drugs.

[114] Tasdemir E., Maiuri M.C., Orhon I., Kepp O., Morselli E., Criollo A., Kroemer G. (2008). p53 represses autophagy in a cell cycle-dependent fashion. Cell Cycle.

[115] Li H., Peng X., Wang Y., Cao S., Xiong L., Fan J., Wang Y., Zhuang S., Yu X., Mao H. (2016). Atg5-mediated autophagy deficiency in proximal tubules promotes cell cycle G2/M arrest and renal fibrosis. Autophagy.

[116] Chen Z.H., Wang W.T., Huang W., Fang K., Sun Y.M., Liu S.R., Luo X.Q., Chen Y.Q. (2017). The lncRNA HOTAIRM1 regulates the degradation of PML-RARA oncoprotein and myeloid cell differentiation by enhancing the autophagy pathway. Cell Death Differ..

[117] Chen Y.-B., Hou J.-H., Feng X.-Y., Chen S., Zhou Z.-W., Zhang X.-S., Cai M.-Y. (2011). Decreased expression of Beclin 1 correlates with a metastatic phenotypic feature and adverse prognosis of gastric carcinomas. J. Surg. Oncol..

[118] Chen N., Karantza-Wadsworth V. (2009). Role and regulation of autophagy in cancer. Biochim. Biophys. Acta Mol. Cell Res..

[119] Karantza-Wadsworth V., Patel S., Kravchuk O., Chen G., Mathew R., Jin S., White E. (2007). Autophagy mitigates metabolic stress and genome damage in mammary tumorigenesis. Genes Dev..

[120] Mathew R., Kongara S., Beaudoin B., Karp C.M., Bray K., Degenhardt K., Chen G., Jin S., White E. (2007). Autophagy suppresses tumor progression by limiting chromosomal instability. Genes Dev..

[121] Edinger A.L., Thompson C.B. (2003). Defective autophagy leads to cancer. Cancer Cell.

[122] Gatica D., Lahiri V., Klionsky D.J. (2018). Cargo recognition and degradation by selective autophagy. Nature.

[123] Poillet-Perez L., Sarry J.-E., Joffre C. (2021). Autophagy is a major metabolic regulator involved in cancer therapy resistance. Cell Rep..

[124] Guo J.Y., Chen H.-Y., Mathew R., Fan J., Strohecker A.M., Karsli-Uzunbas G., Kamphorst J.J., Chen G., Lemons J.M., Karantza V. (2011). Activated Ras requires autophagy to maintain oxidative metabolism and tumorigenesis. Genes Dev..

[125] Lim J., Park H., Heisler J., Maculins T., Roose-Girma M., Xu M., Mckenzie B., van Lookeren Campagne M., Newton K., Murthy A. (2019). Autophagy regulates inflammatory programmed cell death via turnover of RHIM-domain proteins. Elife.

[126] Rangel M., Kong J., Bhatt V., Khayati K., Guo J.Y. (2021). Autophagy and tumorigenesis. FEBS J..

[127] De Souza A.S.C., Gonçalves L.B., Lepique A.P., de Araujo-Souza P.S. (2020). The Role of Autophagy in Tumor Immunology-Complex Mechanisms That May Be Explored Therapeutically. Front. Oncol..

[128] Sheridan M., Ogretmen B. (2021). The Role of Ceramide Metabolism and Signaling in the Regulation of Mitophagy and Cancer Therapy. Cancers.

[129] Xu D.-W., Zhang G.-Q., Wang Z.-W., Xu X.-Y., Liu T.-X. (2015). Autophagy in Tumorigenesis and Cancer Treatment. Asian Pac. J. Cancer Prev..

[130] Zarzynska J.M. (2014). The Importance of Autophagy Regulation in Breast Cancer Development and Treatment. BioMed Res. Int..

[131] Wang H., Yu X., Su C., Shi Y., Zhao L. (2018). Chitosan nanoparticles triggered the induction of ROS-mediated cytoprotective autophagy in cancer cells. Artif. Cells Nanomedicine Biotechnol..

[132] Kim M., Jung J.-Y., Choi S., Lee H., Morales L.D., Koh J.-T., Kim S.H., Choi Y.-D., Choi C., Slaga T.J. (2016). GFRA1 promotes cisplatin-induced chemoresistance in osteosarcoma by inducing autophagy. Autophagy.

[133] Wang Y., Hu Z., Liu Z., Chen R., Peng H., Guo J., Chen X., Zhang H. (2013). MTOR inhibition attenuates DNA damage and apoptosis through autophagy-mediated suppression of CREB1. Autophagy.

[134] Rangwala R., Chang Y.C., Hu J., Algazy K.M., Evans T.L., Fecher L.A., Schuchter L.M., Torigian D.A., Panosian J.T., Troxel A.B. (2014). Combined MTOR and autophagy inhibition: Phase I trial of hydroxychloroquine and temsirolimus in patients with advanced solid tumors and melanoma. Autophagy.

[135] Zhang Q., Wu S., Zhu J., Chai D., Gan H. (2017). Down-regulation of ASIC1 suppressed gastric cancer via inhibiting autophagy. Gene.

[136] Yang S., Lian G. (2019). ROS and diseases: Role in metabolism and energy supply. Mol. Cell. Biochem..

[137] Mandik F., Vos M. (2021). Neurodegenerative Disorders: Spotlight on Sphingolipids. Int. J. Mol. Sci..

[138] Chang K.-C., Liu P.-F., Chang C.-H., Lin Y.-C., Chen Y.-J., Shu C.-W. (2022). The interplay of autophagy and oxidative stress in the pathogenesis and therapy of retinal degenerative diseases. Cell Biosci..

[139] Ornatowski W., Lu Q., Yegambaram M., Garcia A.E., Zemskov E.A., Maltepe E., Fineman J.R., Wang T., Black S.M. (2020). Complex interplay between autophagy and oxidative stress in the development of pulmonary disease. Redox Biol..

[140] Zhang T., Yu J., Cheng S., Zhang Y., Zhou C.-H., Qin J., Luo H. (2023). Research Progress on the Anticancer Molecular Mechanism of Targets Regulating Cell Autophagy. Pharmacology.

[141] Brech A., Ahlquist T., Lothe R.A., Stenmark H. (2009). Autophagy in tumour suppression and promotion. Mol. Oncol..

[142] Liu Y., Levine B. (2014). Autosis and autophagic cell death: The dark side of autophagy. Cell Death Differ..

[143] Li X., He S., Ma B. (2020). Autophagy and autophagy-related proteins in cancer. Mol. Cancer.

[144] Ariosa A.R., Lahiri V., Lei Y., Yang Y., Yin Z., Zhang Z., Klionsky D.J. (2021). A perspective on the role of autophagy in cancer. Biochim. Biophys. Acta Mol. Basis Dis..

